# Enzymatic hydrolysis pretreatment for enhancing the protein solubility and physicochemical quality of *Cordyceps militaris* chicken soup

**DOI:** 10.1002/fsn3.1533

**Published:** 2020-03-25

**Authors:** Hao Dong, Jialing Liu, Xiaofang Zeng, Weidong Bai, Limei Yu

**Affiliations:** ^1^ College of Light Industry and Food Sciences Zhongkai University of Agriculture and Engineering Guangzhou China

**Keywords:** chicken soup, *Cordyceps militaris*, enzymatic hydrolysis, physicochemical quality, protein solubility

## Abstract

Chicken soup is one of the most popular Chinese‐style soups due to its high nutritional value and special flavor. However, the nutrients, mainly soluble protein, in the soup are relatively low. The aim of the present work was to enhance the protein solubility and other physicochemical properties of *Cordyceps militaris* chicken soup by enzymatic hydrolysis pretreatment. Results indicated that the soluble protein dissolution rate and flavor nucleotides (I+G) of *Cordyceps militaris* chicken soup had 1.6‐fold and 0.5‐fold increase, respectively, after enzymatic hydrolysis pretreatment. Not only the contents of total amino acids (TAA) and essential amino acids (EAA) in *Cordyceps militaris* chicken soup significantly increased, the organoleptic quality was also markedly improved after the enzymatic hydrolysis pretreatment. The present work provides a potential approach, which is enzymatic hydrolysis pretreatment of chicken meat, to enhance the protein solubility and physicochemical quality of *Cordyceps militaris* chicken soup.

## Graphical abstract

The originality was to enhance the protein solubility of Cordyceps militaris chicken soup by enzymatic hydrolysis pretreatment with different enzymes. Results indicated that enzymatic hydrolysis pretreatment could significantly enhance the protein solubility, flavor nucleotides (I+G), total amino acid contents, and some other physicochemical properties of Cordyceps militaris chicken soup.

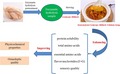

## 1 INTRODUCTION

*Cordyceps militaris* (CM), belonging to *Clavicipitaceae* and *Ascomycotina* families, contains rich amino acids, cordycepin, and polysaccharides (Bi et al., 2018). It has similar pharmacological activities as *Cordyceps sinensis*, a well‐known Chinese traditional medicine (Bai & Sheu, 2018). Extracts of CM which are mainly polysaccharides and cordycepin have been reported to possess a wide range of pharmacological actions such as antimicrobial (Jing et al., 2015), antioxidant (Wu et al., 2014), antitumor (Jin et al., 2018; Liu, Zhu, Sun, Gao, & Zhang, 2017), anti‐inflammatory (Liu et al., 2016), hepatoprotective (Wang et al., 2018), immunostimulatory (Luo et al., 2017), immunomodulatory (Wang et al., 2012), and nephroprotective activities (Chiu et al., 2016; Liu et al., 2015). Considering its various biological activities, CM is widely used for preparation of healthy food. For example, the addition of CM makes chicken soup more helpful for human health (Huang, Tsai, Lee, & Mau, 2006).

Cantonese soup, as one of the most famous Chinese‐style traditional soups, has gained much popularity and acceptance in China and many other countries due to its unique qualities such as flavor, taste, nutrition, and nourishing effect (Qi, Liu, Zhou, & Xu, 2017). As Cantonese soup was usually prepared with a longer stewing time, it is also known as “*Laohuo Tang*.” *Cordyceps militaris* chicken soup which has used chicken and CM as the main materials is one of the most representative Cantonese soups. It is commonly served as a base for savory dishes due to its desirable meaty flavor profile, sweet nature, and delicious taste (Takakura, Mizushima, Hayashi, Masuzawa, & Nishimura, 2014). It is also an excellent soup for persons recovering from illness because it is low in fat; contains rich proteins, free amino acids (FAAs), reducing sugars, and polyunsaturated fatty acids; and is easily digestible (Jayasena et al., 2014).

However, the amount of valuable nutrients such as micronutrients and mainly soluble proteins in soups is relatively small (Jayasena et al., 2015; Qi et al., 2017; Takakura et al., 2014; Zhang et al., 2017). Fortunately, it has been reported that proteins of chicken meat could be hydrolyzed more efficiently via enzymolysis with flavor and neutral protease (Kong, Yang, et al., 2017). In addition, the chicken soup hydrolyzed by enzymes exhibited better taste, quality, and flavor in comparison with traditional chicken soup (Kong, Yang, et al., 2017). In our recently published paper, enzymatic hydrolysis pretreatment was confirmed to be an effective way to enhance volatile flavor compounds of *Cordyceps militaris* chicken soup (Zeng et al., 2020). However, to our best knowledge, studies focused on the enzymatic hydrolysis pretreatment to enhance the protein solubility and physicochemical properties of *Cordyceps militaris* chicken soup have been rarely reported.

Therefore, the main objective of the present study was to enhance the protein solubility and other physicochemical properties of *Cordyceps militaris* chicken soup by enzymatic hydrolysis pretreatment with suitable enzymes. The stewing conditions and the main raw ingredients including chicken and CM were optimized. The results obtained in this work had practical application on quality improvement of *Cordyceps militaris* chicken soup.

## 2 MATERIALS AND METHODS

### 2.1 Materials, chemicals, and reagents

Sanhuang chicken, *Cordyceps militaris*, fresh ginger, and salt**,** which are used for the preparation of soups, were purchased from a Carrefour supermarket in Guangzhou. Flavor protease and compound protease which are used in enzymolysis process were purchased from Novozymes Biotechnology Co. Ltd. HPLC grade of methanol and acetonitrile were purchased from Merck Chemicals Co., Ltd. Other chemicals and reagents were acquired from Guangzhou Chemical Reagent Factory.

### 2.2 Preparation of chicken soups

Various chicken soups were prepared according to procedures reported in our previously published paper (Zeng et al., 2020).

### 2.3 Analysis of crude protein and protein solubility

Crude protein of sample was determined by the Kjeldahl method using a Kjeltec 2300 Analyzer (Foss Tecator). The protein content was obtained by multiplying the total nitrogen value by 6.25 according to previous literature (Wang, Dong, et al., 2016; Wang, He, et al., 2016). The protein solubility was calculated using the following equation:

$$\text{Protein}\;\;\text{solubility}\;\left(\%\right)=100\times P_{1}/P_{2}$$

where the *P*_1_ and *P*_2_ are the values of protein contents (%) of chicken soup powder and its corresponding chicken meat, respectively.

### 2.4 Determination of free amino acid nitrogen content

The free amino acid nitrogen content was measured according to the modified formol titration method described in a reported paper with some modifications (Nilsang, Lertsiri, Suphantharika, & Assavanig, 2005). In brief, 10 g sample was added with an equal amount of distilled water. Then, the mixture was adjusted to pH = 7.0 with 0.1 mol/L NaOH. 10 ml 38% (v/v) formaldehyde solution was subsequently added into the mixture, and titration was performed to the end point at pH = 9.5 with 0.2 mol/L standard NaOH solutions. Three measurements were performed, and the average values were calculated and adopted for each sample.

### 2.5 *Cordyceps* polysaccharides analysis

The *Cordyceps* polysaccharide content was determined on the basis of the phenol–sulfuric acid method described in previous literature with slight modifications (Chen & Huang, 2018). Briefly, the standard curve of polysaccharide was firstly obtained using glucose as the standard and the regression equation was calculated. 10.00 mg sample was accurately weighed and set to 100 ml with distilled water. After adding phenol–sulfuric acid reagent (the distilled water was used as a blank), the mixture was heated in boiling water bath for 10 min and measured at 490 nm with a spectrophotometer (UV2100, UNICO Instrument Co., Ltd.). The determinations were repeated for three times, and the average values were calculated and adopted for each sample.

### 2.6 Amino acid composition analysis

Amino acid composition of sample was analyzed with an amino acid analyzer (L‐8900, Hitachi Co.) according to previous literature (Dong, Zeng, & Bai, 2018; Je, Park, Hwang, & Ahn, 2015). Briefly, 1.0 g sample and 10 ml 6 mol/L HCl were added into an empty tube. Then, the tube was sealed under vacuum and the mixture in which was hydrolyzed at 110°C for 24 hr. 2 ml of hydrolysate at different time intervals of hydrolysis was mixed with equal amount (w/v) of 7% (v/v) 5‐sulfosalicylic acid dehydrate, respectively, and subsequently incubated for 30 min to precipitate protein. The supernatant was then collected by centrifugation at 7511.9 *g*/min for 15 min and analyzed by the amino acid analyzer.

### 2.7 Nucleotide analysis

A Thermo U3000 UPLC System (Thermo Scientific) was adopted for nucleotide analysis according to previous literature (Kong, Yang, et al., 2017; Wang, Dong, et al., 2016; Wang, He, et al., 2016). The nucleotide consisted of 5'‐inosine monophosphate (IMP) and 5'‐guanosine monophosphate (GMP) in the present work. They were detected at wavelength of 254 nm and quantified by the external standard. Nucleotide (I+G) (contents of both IMP and GMP) was calculated, and the average value was adopted.

### 2.8 Organoleptic assessment

The organoleptic assessment was performed by a well‐trained sensory panel which is composed of eight members including four females and four males. All of them had experience in working on food products. The organoleptic assessment took place in a sensory laboratory with international standards according to the method described in previous literature with some modifications (Brückner‐Gühmann, Benthin, & Drusch, 2019; Zhang et al., 2017). Texture, color, taste, aroma, floating oil, and total points were used to describe the organoleptic quality of the tested soups. The intensity of each index was attributed a score from 0 to 10:0 meant “none,” and 10 meant “extremely strong.” During the assessment in the sensory laboratory, 100 ml of soup for each sample was served to each member of the panel in a transparent plastic cup with the constant temperature of 50°C in order to avoid the influence of different temperatures on the flavor attribute. The assessment was performed in triplicate, and the radar maps were obtained using the average values of each index.

### 2.9 Statistical analysis

All data were expressed as means ± *SDs* of three determinations. Statistical calculation and between‐variable correlation were investigated using SPSS (version 12, SPSS Inc.). Significance was determined at *p* < .05 by analysis of variance followed by Duncan's least significant test.

## 3 RESULTS AND DISCUSSION

### 3.1 Selection of chicken portion

**Figure 1:**
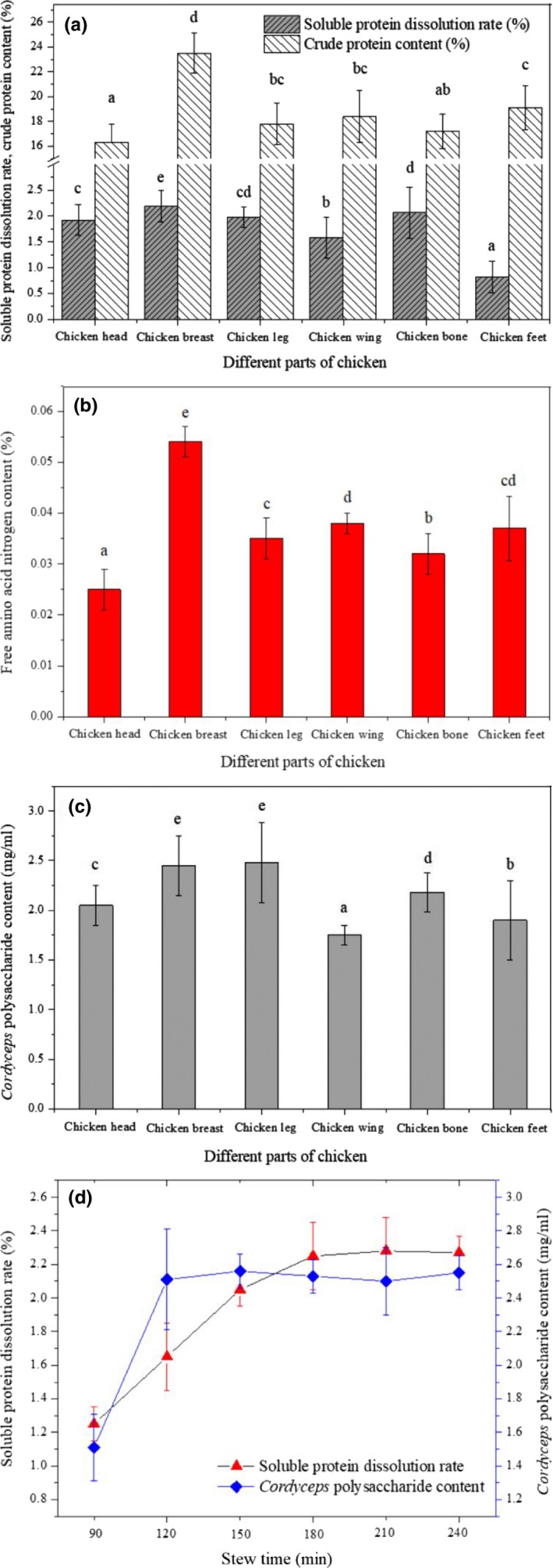
(a–c) Effect of chicken part on the soluble protein dissolution rate, crude protein content, free amino acid nitrogen content, and *Cordyceps* polysaccharide content of chicken soups. (d) Effect of stewing time on the soluble protein dissolution rate and *Cordyceps* polysaccharide content of chicken soups

The chicken portion and stewing conditions used for preparing chicken soup are crucial for releasing flavor compounds and umami taste. Therefore, chicken portion and stewing time were optimized using protein solubility, crude protein content, free amino acid nitrogen content, and *Cordyceps* polysaccharide content by single‐factor experiments. The results are depicted in Figure 1. As shown in Figure 1a, chicken breast had the highest crude protein content (23.35%), which was significantly higher (*p* < .05) than those of other chicken parts. The soluble protein dissolution rates of chicken soups were remarkably (*p* < .05) affected by the chicken parts used. The soluble protein dissolution rate in chicken soup prepared with chicken breast had the highest value (2.18%), probably due to the highest crude protein content in chicken breast. As for free amino acid nitrogen content (Figure 1b), significant differences (*p* < .05) were found among six groups and the highest value was also recorded in chicken soup prepared with chicken breast. This result could also attribute to the highest crude protein content in chicken breast. The free acid nitrogen content was an indicator of the cleavage of peptide bonds, and the increase of which indicated high MW proteins were degraded into small MW proteins or peptides during heating process (Wang, Dong, et al., 2016; Wang, He, et al., 2016). Protein hydrolysates rich in low MW peptides have more dietary uses because of higher therapeutic and nutritional values, which can improve nutritional value of chicken soup (Bhaskar, Modi, Govindaraju, Radha, & Lalitha, 2007).

No significant differences (*p* > .05) were observed between *Cordyceps* polysaccharide contents of chicken soups prepared with chicken breast and chicken leg, while significant differences (*p* < .05) were found among the rest groups (Figure 1c). Recent investigations have shown that *Cordyceps* polysaccharide possesses various biological activities including antioxidation, immunomodulation, antitumor, and anti‐inflammation (Jing et al., 2015; Wang et al., 2012). It is worth mentioning that *Cordyceps* polysaccharide contents of chicken soups prepared with chicken breast and chicken leg are significantly higher (*p* < .05) than those of the rest groups, which can significantly enhance the biological activities of chicken soup. In conclusion, chicken breast was selected to prepare chicken soup.

Free amino acid compositions in chicken soups prepared with different portions of chicken (%) are presented in Table 1. Sixteen free amino acids were detected in all chicken soups, and the predominant free amino acids were Gly and Glu. In contrast, Glu and Ser were reported to be the predominant free amino acids in chicken broth cube, and Lys, Glu, Ala, and Ser were the major free amino acids in yellow‐feather chicken soup (Li et al., 2018). The composition of most free amino acids in chicken soups differed significantly between the chicken parts selected. Different raw materials used to produce chicken soups might result in variations in the free amino acid compositions (Jayasena et al., 2014; Xian et al., 2019). Among the 16 amino acids, Asp and Glu are associated with a umami flavor (Kong, Yang, et al., 2017). They were significantly higher (17.50%) in soup prepared with chicken breast compared with those prepared with other chicken parts, leading to a better flavor of chicken soup. According to FAO and WHO, foods with EAA/TAA value about 40% and EAA/NEAA value beyond 60% are ideal protein sources (Jiang & Nie, 2015). EAA/TAA and EAA/NEAA values of chicken soup prepared with chicken breast were also more close to the specified values compared with the rest soups.

**Table 1:** Amino acid composition in *Cordyceps militaris* chicken soups prepared with different parts of chicken (%)

Amino acids	Heads	Breasts	Legs	Wings	Bones	Feet
Met^a^	0.45	0.76	0.50	0.59	0.58	0.89
Val^a^	2.87	3.03	2.75	2.59	2.64	2.32
Leu^a^	4.80	3.79	4.62	4.00	4.25	3.93
Phe^a^	4.36	13.64	5.50	6.36	6.32	3.21
Ile^a^	1.73	2.20	1.65	1.46	1.55	1.54
Thr^a^	3.07	3.86	5.11	4.55	5.06	2.68
Lys^a^	5.94	5.99	6.04	5.00	5.40	4.82
Glu	11.39	13.64	12.64	10.00	11.49	9.46
Asp	3.56	3.86	3.68	3.55	3.28	3.39
Gly	24.75	9.85	24.73	24.09	22.99	27.86
Ser	3.32	3.64	2.97	2.82	2.93	2.86
Ala	10.89	6.36	11.54	10.46	10.35	10.71
His	1.39	18.94	3.08	3.41	3.16	0.95
Arg	9.41	5.08	9.34	8.64	8.62	10.00
Pro	10.89	3.64	10.44	11.26	9.77	14.29
Tyr	1.44	2.05	1.37	1.09	1.32	1.00
EAA/TAA, %	23.22	29.39	26.15	24.55	25.81	19.39
EAA/NEAA, %	30.24	41.63	35.41	32.54	34.78	24.05

Note

EAA, NEAA, and TAA are abbreviations for essential amino acids, nonessential amino acids, and total amino acids, respectively. EAA were calculated as the total of Thr, Val, Lys, Met, Ile, Phe, and Leu.

^a^ Means essential amino acids.

### 3.2 Optimization of stewing time

From Figure 1d, a significant increase (*p* < .05) in soluble protein dissolution rate was observed with the strewing time being increased from 90 to 180 min, and no significant difference (*p* > .05) was observed with further increase in strewing time. This result was in agreement with that in a previous literature (Qi et al., 2017). It also reported that more fat‐ and water‐soluble compounds (fat, proteins, nucleotides, organic acids, and free amino acids) transferred into the chicken soup from the chicken meat with the prolonged stewing time (1–3 hr) (Qi et al., 2017). The *Cordyceps* polysaccharide content was also significantly increased (*p* < .05) from 90 to 120 min, and no significant differences (*p* > .05) were observed with further increase from 120 to 240 min. In the heating process, the *Cordyceps* polysaccharide dissolved quickly in chicken soup at the beginning. However, with the increase in stewing time, *Cordyceps* polysaccharide was dissolved more fully, leading to its saturation in chicken soup. Therefore, stewing time of 180 min was finally selected.

### 3.3 Optimization of enzymatic hydrolysis conditions

**Figure 2:**
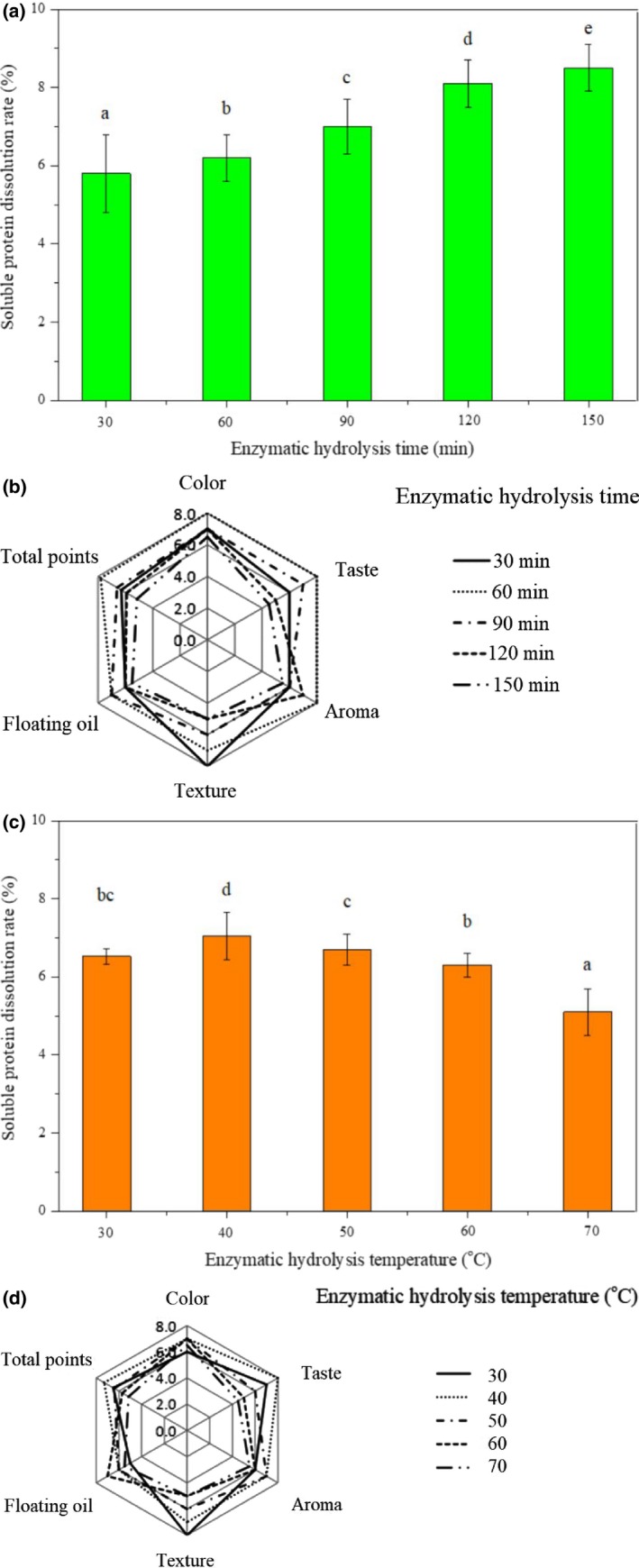
Effect of enzymolysis time on the soluble protein dissolution rate (a) and organoleptic quality (b) of chicken soups. Effects of enzymolysis temperature on the soluble protein dissolution rate (c) and organoleptic quality (d) of chicken soups

Enzymatic hydrolysis is an effective method for the recovery of proteins, and protein hydrolysates have good physicochemical properties (Wang, Dong, et al., 2016; Wang, He, et al., 2016). The protein solubility and organoleptic assessment were selected to optimize the enzymolysis conditions. As shown in Figure 2a, the protein solubility increased significantly (*p* < .05) as the enzymatic hydrolysis time increased from 30 min to 150 min. However, when the enzymatic hydrolysis time was longer than 90 min, the chicken meat became rotten and had bad taste (Figure 2b). Overall, the enzymatic hydrolysis time of 60 min was selected due to the strong fragrance, delicate taste, and good color of chicken soup. The protein solubility increased significantly (*p* < .05) with the enzymatic hydrolysis temperature being increased from 30 to 40°C; then, significant decreases (*p* < .05) were observed when the enzymatic hydrolysis temperature continually increased from 40 to 70°C (Figure 2c). This was because the enzyme activity of proteases decreased at higher temperatures, resulting in insufficient enzymatic hydrolysis between chicken and complex enzymes and decrease in the soluble protein content. Moreover, the chicken meat became rotten, the soup color was cloudy, and there was almost no oil on the surface of chicken soup when enzymatic hydrolysis temperature was higher than 50°C (Figure 2d). Hence, 40°C was chosen as the enzymatic hydrolysis time.

### 3.4 Physicochemical changes of *Cordyceps militaris* chicken soups

The free amino acid profiles, the main physicochemical changes, and sensory quality scores of *Cordyceps militaris* chicken soups with and without the optimized enzymatic hydrolysis pretreatment are presented in Tables 2, 3, 4, respectively. As shown in Table 2, the total free amino acids increased from 1,320 mg/100 g of *Cordyceps militaris* chicken soup to 3,330 mg/100 g of the enzymolysis *Cordyceps militaris* chicken soup. The source of FAA in the soup is possibly associated with two ways: one is the migration of the original free amino acid from the chicken meat into soup, and the other is the degradation of proteins and peptides (Zhang et al., 2013). Enzymatic hydrolysis pretreatment can enhance the degradation of proteins and peptides in chicken meat, resulting in remarkable increase in free amino acids. Free amino acids are vital for flavor development and therefore for enhancing edible value of meat and fish (Dong et al., 2020; Feng, Zhu, Liu, Lai, & Yang, 2017; Jayasena et al., 2015). FAAs associated with taste components can be classified into four classes: umami, sweet, bitter, and tasteless. Asp and Glu are umami taste, Gly, Ala, Ser, Pro, and Thr are sweet taste, while Val, Met, Ile, Phe, Lys, Leu, Arg, His, and Tyr are bitter taste, and cysteine is identified as tasteless amino acid (Kong, Yang, et al., 2017; Kong, Zhang, et al., 2017). From Table 2, enzymolysis *Cordyceps militaris* chicken soup had a significantly higher (*p* < .05) level of umami amino acids (Glu and Asp) than that from *Cordyceps militaris* chicken soup. The flavor nucleotides mainly include 5 '‐IMP and 5'‐GMP, which also have the function of umami taste (Phat, Moon, & Lee, 2016). From Table 3, nucleotide (I+G) content also significantly increased after the enzymatic hydrolysis pretreatment, from 19.21 to 28.38 mg/100 ml. Contents of FAAs responsible for sweet and bitter taste increased approximately twofold and onefold, respectively, after enzymatic hydrolysis pretreatment. Surprisingly, cysteine was absent in all chicken soups. This result was in accordance with Jayasena et al. (2014) who also found the absence of cysteine in freeze‐dried chicken soups. It was attributed to the fact that the available cysteine might have been utilized completely for flavor development during preparation of chicken soups (Jayasena et al., 2014).

**Table 2:** Amino acid profiles of *Cordyceps militaris* chicken soup and enzymolysis *Cordyceps militaris* chicken soup

Amino acids	*Cordyceps militaris* chicken soup		Enzymolysis *Cordyceps militaris* chicken soup	
	Free amino acids (mg/100 g)	Relative content (%)	Free amino acids (mg/100 g)	Relative content (%)
Glu	180.00	13.64	550.00	16.52
Asp	51.00	3.86	240.00	7.21
Gly	130.00	9.85	200.00	6.01
Ser	48.00	3.64	150.00	4.51
Ala	84.00	6.36	230.00	6.91
Met^a^	10.00	0.76	50.00	1.50
Val^a^	40.00	3.03	140.00	4.20
Leu^a^	50.00	3.79	240.00	7.21
His	250.00	18.94	310.00	9.31
Arg	67.00	5.08	230.00	6.91
Phe^a^	180.00	13.64	240.00	7.21
Ile^a^	29.00	2.20	130.00	3.90
Thr^a^	51.00	3.86	140.00	4.20
Pro	48.00	3.64	84.00	2.52
Tyr	27.00	2.05	78.00	2.34
Lys^a^	79.00	5.99	320.00	9.61
TAA	1,320	‐	3,330.00	‐
Umami amino acids	231.00	17.50	790.00	23.72
Sweet amino acids	313.00	23.64	720.00	21.62
Bitter amino acids	536.00	40.48	998.00	29.97
EAA	388.00	23.26	1,260.00	37.84
EAA/TAA, %	‐	29.39	‐	37.84
EAA/NEAA, %	‐	41.63	‐	60.87

Note

EAA, NEAA, and TAA are abbreviations for essential amino acids, nonessential amino acids, and total amino acids, respectively.

^a^ Means essential amino acids.

**Table 3:** Differences of several physicochemical parameters between *Cordyceps militaris* chicken soup and enzymolysis *Cordyceps militaris* chicken soup

Index	*Cordyceps militaris* chicken soup	Enzymolysis *Cordyceps militaris* chicken soup	Multiples (the latter/former)
Soluble protein dissolution rate, %	3.16 ± 0.24	8.23 ± 0.46	2.6
Flavor nucleotides (I + G), mg/100 ml	19.21 ± 2.12	28.34 ± 1.56	1.5
Total amino acid contents, mg/100 g	1,320 ± 140	3,330 ± 205	2.5
Essential amino acid relative contents, mg/100 g	388 ± 75	1,260 ± 122	3.2

**Table 4:** Sensory quality scores of *Cordyceps militaris* chicken soup and enzymolysis *Cordyceps militaris* chicken soup

Index	Color	Taste	Aroma	Texture	Floating oil	Total points
*Cordyceps militaris* chicken soup	7.3 ± 0.56^a^	7.1 ± 0.43^a^	7.2 ± 0.34^a^	7.7 ± 0.32^a^	8.1 ± 0.44^a^	7.3 ± 0.19^a^
Enzymolysis *Cordyceps militaris* chicken soup	7.9 ± 0.56^b^	8.6 ± 0.39^b^	8.1 ± 0.39^a^	7.1 ± 0.25^b^	8.6 ± 0.41^a^	8.2 ± 0.32^b^

Note

Values in a row followed by the different letters are significantly different (P < 0.05).

The essential amino acid (EAA) content in chicken soup increased significantly (*p* < .05) with the enzymatic hydrolysis pretreatment, from 388 to 1,260 mg/100 g. The EAA/TAA and EAA/NEAA values also increased from 29.39% to 37.84% and 41.63 to 60.87%, respectively (Tables 2 and 3), which were all comparative to the values specified by FAO and WHO for ideal protein sources (Jiang & Nie, 2015). In addition, the soluble protein dissolution rate of enzymolysis *Cordyceps militaris* chicken soup was 2.6 times of that of *Cordyceps militaris* chicken soup (Table 3). This is because that enzymatic hydrolysis can effectively degrade proteins and enhance the protein solubility of chicken soup. The total score of *Cordyceps militaris* chicken soup prepared with enzymatic hydrolysis pretreatment was higher than that of *Cordyceps militaris* chicken soup (Table 4). The taste and aroma were more mellow and sweet after the enzymatic hydrolysis pretreatment. However, the tissue status of enzymolysis *Cordyceps militaris* chicken soup was inferior to that of *Cordyceps militaris* chicken soup probably due to the soft texture of chicken meat resulted from enzymatic hydrolysis.

## 4 CONCLUSION

In order to enhance protein solubility and physicochemical quality of *Cordyceps militaris* chicken soup, a pretreatment of enzymatic hydrolysis of chicken meat was performed. Results indicated that the enzymatic hydrolysis pretreatment significantly enhanced the protein solubility and some other parameters of *Cordyceps militaris* chicken soup without destroying its organoleptic quality. Enzymatic hydrolysis of chicken is confirmed to be an effective method to enhance the protein solubility and physicochemical quality of *Cordyceps militaris* chicken soup.

## CONFLICT OF INTEREST

All authors declare that they have no conflict of interest.

## ETHICAL APPROVAL

This article does not contain any studies with human participants or animals performed by any of the authors.
